# Why do snails have hairs? A Bayesian inference of character evolution

**DOI:** 10.1186/1471-2148-5-59

**Published:** 2005-11-04

**Authors:** Markus Pfenninger, Magda Hrabáková, Dirk Steinke, Aline Dèpraz

**Affiliations:** 1 Abteilung Ökologie & Evolution, J.W. Goethe-Universität, BioCampus Siesmayerstraße, 60054 Frankfurt/Main, Germany; 2 Deparment of Zoology, Charles University, Viniènà 7, 128 44 Praha 2, Czech Republic; 3 Department of Biology, University of Konstanz, Postbox 5560 M618, 78457 Konstanz, Germany; 4 Département d'Ecologie et Evolution, Université de Lausanne, Bâtiment de Biologie, Dorigny, 1015 Lausanne, Switzerland

## Abstract

**Background:**

Costly structures need to represent an adaptive advantage in order to be maintained over evolutionary times. Contrary to many other conspicuous shell ornamentations of gastropods, the haired shells of several Stylommatophoran land snails still lack a convincing adaptive explanation. In the present study, we analysed the correlation between the presence/absence of hairs and habitat conditions in the genus *Trochulus *in a Bayesian framework of character evolution.

**Results:**

Haired shells appeared to be the ancestral character state, a feature most probably lost three times independently. These losses were correlated with a shift from humid to dry habitats, indicating an adaptive function of hairs in moist environments. It had been previously hypothesised that these costly protein structures of the outer shell layer facilitate the locomotion in moist habitats. Our experiments, on the contrary, showed an increased adherence of haired shells to wet surfaces.

**Conclusion:**

We propose the hypothesis that the possession of hairs facilitates the adherence of the snails to their herbaceous food plants during foraging when humidity levels are high. The absence of hairs in some *Trochulus *species could thus be explained as a loss of the potential adaptive function linked to habitat shifts.

## Background

Evolutionary theory predicts that costly structures must convey a fitness advantage to their bearers in order to be maintained over evolutionary time [1]. Flightlessness in birds and insects, limblessness in lizards and sightlessness in cave-dwelling organisms are some prominent examples of phenotypic regression due to the loss of adaptive function (reviewed in [2]). Molluscs in general and gastropods in particular display a fascinating diversity of elaborate shell structures [3,4] and have attracted considerable research efforts to explain them in adaptive terms [5-7]. The proposed roles invoked mechanical stability [8], defence against predators [9], sexual selection [10] and climatic selection [11]. However, the potential selective advantage of hair-like shell ornamentation of certain land snail species remains unknown.

These hairs can reach varying densities (up to 20 per squaremilimetre) and lengths (up to three millimetres). In some cases hardly visible, they confer an almost furry impression to the shell in others. These semi-rigid structures are part of the periostracum, a thin protein layer (conchiolin) secreted by the snail to cover the calcareous shell [12]. Building hairs requires the snail to have specialised glandular tissue and complex strategies to form them. Consequently, this trait can be assumed to be costly and should thus present a selective advantage to its bearers in order to be conserved.

Haired shells occur in several species of the Stylommatophoran families Polygyridae, Helicidae and Hygromiidae. These families are only distantly related [13], suggesting that this features has evolved several times independently. Haired shells are almost exclusively observed in species living in moist microhabitats, like layers of fallen leaves, broad-leaved vegetation, damp meadows or wet scree [14]. Such a correlation suggests an adaptive significance of the trait in such a habitat [1]; it was thus speculated that the hygrophobic hairs facilitate the movement in wet environments by relieving surface tension [14,15]. A correlation between haired shells and humid habitats is thus expected. In order to test this, we employed the recent Bayesian extensions of the comparative method, allowing to take mapping and phylogenetic uncertainty simultaneously into account [16]. With a diversity hotspot in South Germany, Eastern France and Switzerland, the land snail genus *Trochulus s. str*. (common name: Hairy snails) is particularly suited to address our question: its species exhibit variability in both hairiness and ecology. This study present the first comprehensive molecular phylogeny for the genus *Trochulus *Chemnitz, 1786 (until recently *Trichia*, Hartmann 1840) based on mitochondrial and nuclear loci. Finally, we tested experimentally whether the possession of haired shells indeed facilitates locomotion.

## Results

### Lineage identification and phylogenetic relations

**Figure 1:**
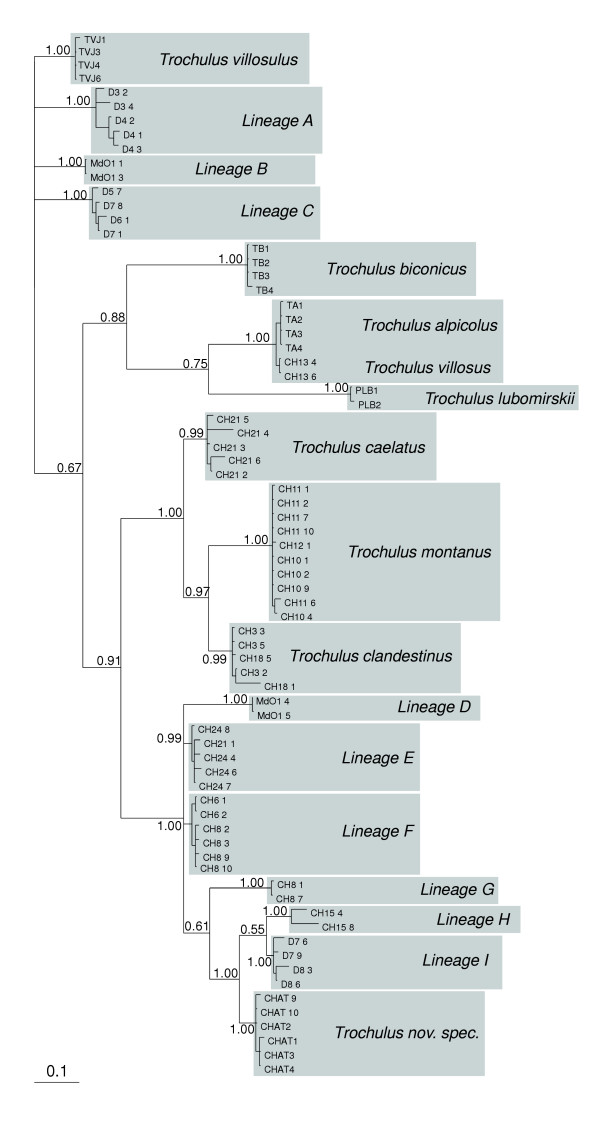
**Unrooted consensus tree of 90,000 trees sampled by the Markov-chain in Bayesian analysis for the COI-fragment**. Numbers on nodes indicate the Bayesian posterior probability.

The initial phylogenetic analysis on a COI data set of the presumed *Trochulus *species resolved 18 terminal clades, each with 0.99 posterior probabilities or higher (Figure 1). The uncorrected sequence divergence among those clades ranged from 0.029 to 0.173 (Table 2). Out of these lineages, nine could be assigned to existing taxa, because the species were sampled from the type locality and/or were morphologically unmistakable. The nine remaining clades, however, could not be unequivocally attributed to a taxonomic name. All eighteen identified lineages were used as molecularly defined operational taxonomic units in the subsequent analyses [17].

**Table 2:** Pairwise uncorrected COI sequence divergence among lineages and species (mean ± s.d.).

	*A*	*B*	*C*	*villosulus*	*montanus*	*clandest.*	*caelatus*	*D*	*E*	*F*	*G*	*H*	*I*	*biconicus*	*nov. spec.*	*villosus*
*B*	*0.111 ± 0.015*		*striolatus/plebeius*													
*C*	*0.118 ± 0.015*	*0.091 ± 0.013*	clade													
*villosulus*	*0.104 ± 0.014*	*0.073 ± 0.013*	*0.091 ±* *0.014*													
*montanus*	0.143 ± 0.017	0.144 ± 0.017	0.138 ± 0.017	0.117 ± 0.016												
*clandestinus*	0.135 ± 0.017	0.129 ± 0.016	0.140 ± 0.017	0.125 ± 0.016	*0.083 ± 0.014*	Jura clade										
*caelatus*	0.144 ± 0.016	0.152 ± 0.018	0.141 ± 0.017	0.130 ± 0.016	*0.092 ± 0.014*	*0.093 ± 0.013*										
*D*	0.146 ± 0.017	0.105 ± 0.016	0.125 ± 0.016	0.127 ± 0.017	0.142 ± 0.017	0.126 ± 0.016	0.138 ± 0.017									
*E*	0.148 ± 0.017	0.109 ± 0.016	0.135 ± 0.016	0.117 ± 0.016	0.134 ± 0.016	0.104 ± 0.014	0.107 ± 0.015	*0.079 ± 0.013*						*sericeus/hispidus*		
*F*	0.152 ± 0.017	0.112 ± 0.015	0.126 ± 0.015	0.119 ± 0.016	0.146 ± 0.016	0.113 ± 0.015	0.114 ± 0.015	*0.072 ± 0.013*	*0.029 ± 0.008*					clade		
*G*	0.142 ± 0.017	0.139 ± 0.017	0.136 ± 0.018	0.109 ± 0.016	0.132 ± 0.017	0.117 ± 0.016	0.147 ± 0.016	*0.120 ± 0.017*	*0.111 ± 0.016*	*0.105 ± 0.015*						
*H*	0.164 ± 0.017	0.153 ± 0.018	0.170 ± 0.017	0.169 ± 0.018	0.162 ± 0.017	0.126 ± 0.015	0.147 ± 0.017	*0.116 ± 0.015*	*0.100 ± 0.014*	*0.101 ± 0.014*	*0.128 ± 0.016*					
*I*	0.150 ± 0.017	0.151 ± 0.017	0.155 ± 0.017	0.144 ± 0.017	0.148 ± 0.016	0.124 ± 0.015	0.133 ± 0.016	*0.106 ± 0.014*	*0.102 ± 0.014*	*0.099 ± 0.013*	*0.106 ± 0.014*	*0.061 ± 0.010*				
*biconicus*	0.158 ± 0.018	0.136 ± 0.017	0.141 ± 0.018	0.128 ± 0.018	0.164 ± 0.020	0.154 ± 0.018	0.167 ± 0.019	*0.145 ± 0.017*	*0.145 ± 0.018*	*0.145 ± 0.018*	*0.143 ± 0.018*	*0.179 ± 0.018*	*0.167 ± 0.017*			
*nov. spec.*	0.153 ± 0.017	0.137 ± 0.017	0.151 ± 0.017	0.142 ± 0.017	0.151 ± 0.016	0.128 ± 0.015	0.137 ± 0.016	*0.100 ± 0.015*	*0.082 ± 0.014*	*0.081 ± 0.013*	*0.106 ± 0.015*	*0.080 ± 0.013*	*0.057 ± 0.011*	*0.161 ±* *0.018*		
*villosus*	0.144 ± 0.018	0.139 ± 0.018	0.154 ± 0.018	0.152 ± 0.018	0.160 ± 0.018	0.153 ± 0.018	0.160 ± 0.018	0.168 ± 0.019	0.149 ± 0.018	0.151 ± 0.018	0.173 ± 0.019	0.171 ± 0.019	0.165 ± 0.018	0.163 ± 0.019	0.168 ± 0.019	*villosa/alpicola*
*alpicolus*	0.142 ± 0.016	0.141 ± 0.015	0.151 ± 0.018	0.153 ± 0.017	0.159 ± 0.014	0.151 ± 0.019	0.162 ± 0.019	0.167 ± 0.018	0.147 ± 0.016	0.150 ± 0.019	0.173 ± 0.019	0.173 ± 0.020	0.162 ± 0.016	0.161 ± 0.020	0.170 ± 0.020	*0.006 ±* *0.004*

**Figure 2:**
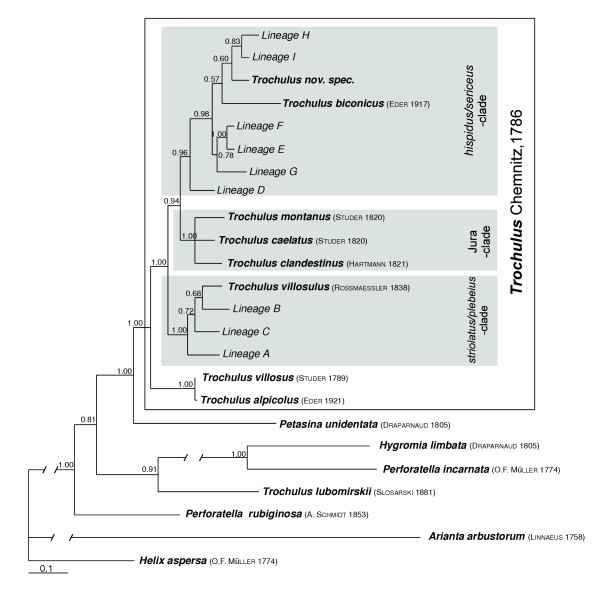
**Consensus tree of 90,000 trees sampled by the Markov-chain in Bayesian analysis for the total data set (1383 bp of COI, 16S and ITS1)**. Numbers on nodes indicate the Bayesian posterior probabilities.

The Bayesian phylogenetic analysis of the entire data set (COI, 16S and ITS-1) showed the monophyly of the genus *Trochulus *within the Hygromiinae with high posterior probability, except for *T. lubomirskii*, which seems to be only distantly related to this genus (Figure 2). In addition to the early branching *T. villosus/alpicolus *clade, the genus is composed of three well supported subclades: first, a clade containing the *T. striolatus/plebeius*-like lineages together with *T. villosulus*, a second clade with ecologically divers species confined to the Jura mountains and the neighbouring Mittelland-plain and finally, a *T. hispidus/sericeus*-like clade, containing also *T. biconicus *and a new species.

### Correlation of shell hairiness with habitat

**Figure 3:**
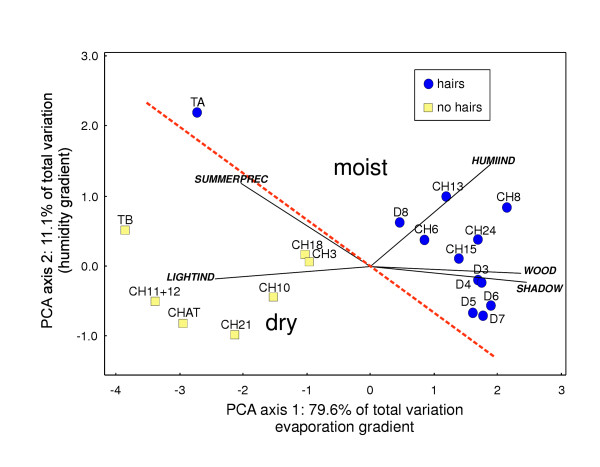
**Two first components of the PCA of the sampled localities over 9 environmental variables**. Black dots: populations with haired individuals. Open squares: populations with hair-less individuals. Sampling sites above the dotted line are considered moist whereas those under it are dry.

The PCA on habitat humidity describing variables resulted in two meaningful axes, representing 79.7% and 13.4% of the total variation. The first component opposed sampling sites in shady woods and sites in sun exposed, open areas. This axis can therefore be interpreted as an evaporation gradient. The second axis is a gradient of the summer precipitation on one hand and the humidity demand of the vegetation on the other (Figure 3). It can thus be considered as a humidity gradient. The sampling sites appear as two distinct clusters that could be classified as either moist or dry (Table 1). The outlier (TA) was also considered to be humid, according to its high humidity levels. For each population, at least ten adult individuals were scored for the presence or absence of hairs (mixed populations were not found). Non-haired populations exclusively corresponded to species described in the literature as having smooth shells (Table 1). When plotting the hairiness of each population on the PCA, a complete congruence between humidity and hairiness became apparent: haired shells tended to occur at sites with low evaporation and/or high precipitation while smooth shells were found at places with high evaporation and/or lower precipitation (Figure 3).

**Table 1:** Table of sampling sites, presumed taxon, habitat characterisation and presence or absence of hairs.

Sampling site	Abbreviation	Geographical position	Presumed taxon	Habitat	Humidity	Hairs
Burgsinn, Bayern, Germany	D3	50°09' 31"N 09°40'34"E	*T. striolatus/plebeius*	wood	moist	yes
Habichtstal, Bayern, Germany	D4	50°02'54"N 09°25'41"E	*T. striolatus/plebeius*	wood	moist	yes
Dommershausen, Rheinland-Pfalz, Germany	D5	50°07'45"N 07°23'47"E	*T. striolatus/plebeius*	wood	moist	yes
Bingen, Rheinland-Pfalz, Germany	D6	49°55'56"N 07°58'57"E	*T. striolatus/plebeius*	wood	moist	yes
Eltville, Hessen, Germany	D7	50°0059"N 08°04'28"E	*T. striolatus/plebeius T. sericeus/hispidus*	wood	moist	yes
Büchsenberg, Baden-Württemberg, Germany	D8	48°04'55"N 07°37'23"E	*T. striolatus/plebeius*	wood	moist	yes
St. Seine l'Abbaye, Côte d'Or, France	MdO	47°26'04"N 04°46'55"E	*T. sericeus/hispidus*	wood	-	yes
La Neirigue, Fribourg, Switzerland	CH3	46°42'16"N 06°55'12"E	*T. clandestinus*	riverbank vegetation	moist	no
Barrage des Rossens, Fribourg, Switzerland	CH6	46°43'33"N 07°06'55"E	*T. sericeus/hispidus*	wood	moist	yes
Gorges de la Jogne, Fribourg, Switzerland	CH8	46°36'45"N 07°07'10"E	*T. sericeus/hispidus*	gorge	moist	yes
Ste Croix, Vaud, Switzerland	CH10	46°50'44"N 06°32'02"E	*T. montanus*	open wood	dry	no
La Côte aux Fées, Neuchâtel, Switzerland	CH11+12	46°50'74"N 06°32'42"E	*T. montanus*	grassland	dry	no
Col des Mosses, Vaud, Switzerland	CH13	46°25'49"N 07°08'22"E	*T. villosus*	wood	moist	yes
Vallée du Rhône, Vaud, Switzerland	CH15	46°19'43"N 06°13'39"E	*T. sericeus/hispidus*	wood	moist	yes
Sensetal, Bern, Switzerland	CH18	46°49'46"N 07°19'19"E	*T. clandestinus*	riverbank vegetation	dry	no
Birseschlucht, Bern, Switzerland	CH21	47°17'54"N 07°23'00"E	*T. caelatus*	cliff	dry	no
Birseschlucht, Bern, Switzerland	CH24	47°16'56"N 07°23'13"E	*T. sericeus/hispidus*	wood	moist	yes
Château d'Oex, Vaud, Switzerland	CHAT	46°16'23"N 07°21'42"E	*T. nov. spec.*	alpine meadow	dry	no
Bannalppass, Nidwalden, Switzerland	TA	46°53'40"N 08°27'15"E	*T. alpicolus*	alpine meadow	moist	yes
Bannalppass, Nidwalden, Switzerland	TB	46°53'43"N 08°27'21"E	*T. biconicus*	alpine meadow	dry	no
Velká Javořina, Velká nad Veličkou, Czech Republic	TVJ	48°51'26"N 17°39'11"E	*T. villosulus*	wood	moist	yes
Bohuslavice u Zlína, Czech Republic	PLB	49°09'19"N 17°37'29"E	*T. lubomirskii*	meadow	-	yes

### Character state evolution

**Figure 4:**
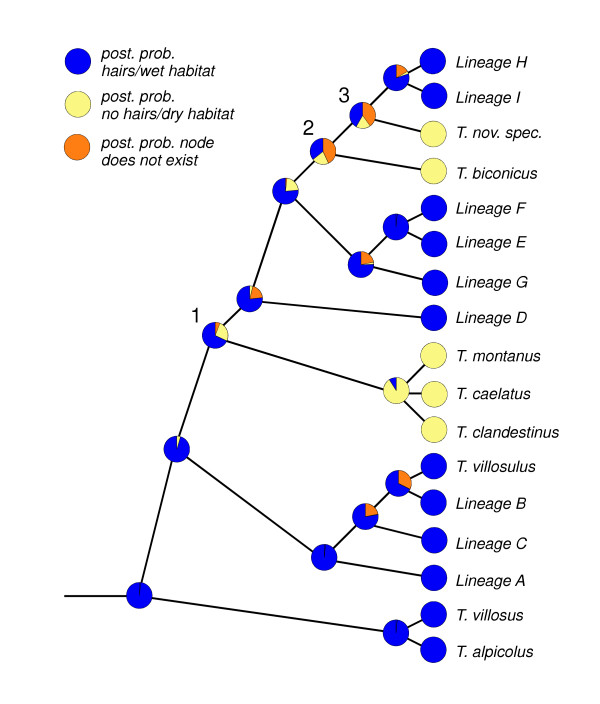
Bayesian reconstruction of ancestral states (hairs/no hairs, moist/dry habitat, respectively) on the topology of the Bayesian consensus tree (restricted to the *Trochulus*-clade).

As the occurrence in moist habitats was systematically linked to the presence of hairs in *Trochulus s.str*., only a single analysis was necessary for both characters. The Bayesian analysis of character evolution suggested with high posterior probability that the most recent common ancestor of the genus *Trochulus *most likely possessed hairs and lived in a moist habitat (Figure 4). The analysis also revealed considerable mapping- and/or phylogenetic uncertainty in the reconstruction of crucial ancestral nodes (nodes 1–3 in Figure 4). The average Bayesian parameter estimate for the character change ratio was 2.50 ± 0.11 (mean ± s.d.), indicating that a loss of hairs associated with a transition from wet to dry habitats occurred more frequently than vice versa. This was in concordance with the parsimony reconstruction of character state changes on all different topologies of the 99% credibility set of trees. A minimum number of three independent losses of hairs / habitat transitions had a higher probability (0.59) than the only other observed pattern of two losses/one gain or three losses/no gain (0.41).

### Functional analysis

**Figure 5:**
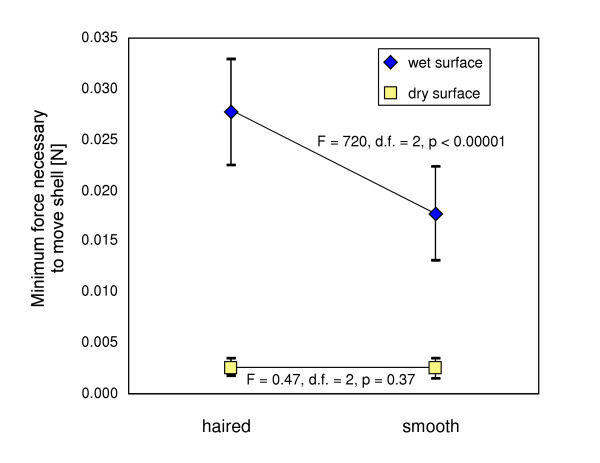
Mean (+/- s.d.) minimum necessary force to move haired and smooth shells on wet and dry leaf surfaces.

The analysis of variance showed that on a water-covered leaf surface, hairy shells required a significantly higher minimum force to overcome the adhesion (F = 720, d.f. = 2, p < 0.00001). There was no difference on a dry surface (F = 0.47, d.f. = 2, p = 0.37; Figure 5).

## Discussion

Considering the limited number of sites sampled, we found a relatively large number of lineages, most of which could not be attributed to described species. This suggests that many other more or less morphologically similar entities may exist throughout the range of the genus. The existence of cryptic lineages could explain at least in part the current taxonomic uncertainty in *Trochulus *[18-20]. For example, several subspecies have been described for *T. striolatus *[21], which may well represent distinct evolutionary lineages such as described here. Given that the sequence divergence among the nine unidentified lineages is of the same magnitude as among described, morphologically and ecologically distinct species (Figure 1, Table 2), it can be reasonably assumed that the cryptic lineages within the *striolatus/plebeius *and *hispidus/sericeus *clades correspond to good species. Even under the assumption of an exceptionally fast molecular clock in land snails of up to 5% sequence divergence per one million years [22], the lineages in the *striolatus/plebeius *clade, for example, persisted for at least two million years as independent evolutionary entities. The existence of more or less cryptic lineages or species is not an unusual finding in land snails [23-25]. In contrast to the high divergence of the unidentified lineages, the comparatively small genetic distance between *T. villosus *and *T. alpicolus *indicated a questionable specific distinction between these two taxa. Detailed phylogeographic analyses in addition to morphometric and ecological studies will be necessary to disentangle the species limits of these cryptic *Trochulus *complexes, clarify the taxonomy and reveal their evolutionary history. In addition, the species *T. lubomirskii*, which was placed by Schileyko [26] into the subgenus *Plicuteria*, may not belong to the genus *Trochulus *at all.

A haired shell appears as the ancestral state in the genus *Trochulus*. This inference is strengthened by the observation that some of the hair-less species do possess some as juveniles. During the evolutionary history of the genus *Trochulus*, hairs appear to have been lost several times independently (Fig. 3, Table 1) and this was always correlated with a shift in habitat (i.e. hairs are only present in moist habitats, mostly woodlands). This suggests that hairs potentially have an adaptive function in humid habitats and once the presumed selective pressure for the maintenance of these costly protein structures is relieved, they are lost. Such a correlation makes certain potential adaptive explanations for hairiness unlikely: defence against predators or mechanical stability have no obvious reasons to co-vary with the humidity characteristics of a habitat.

**Figure 6:**
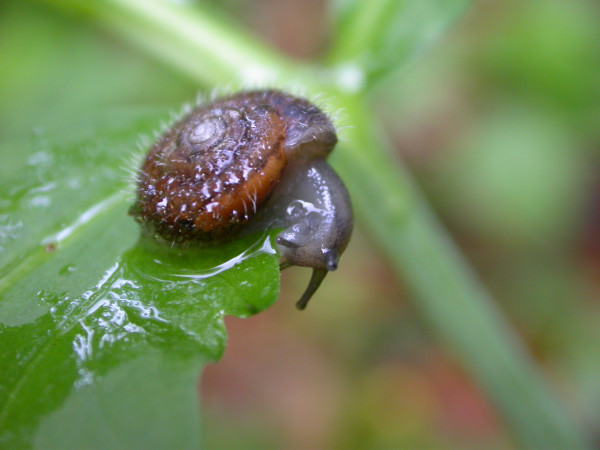
**Active *T. villosus *foraging on a leaf**. Note that the water-film on the leaf is adhering to the shell.

The facilitation of locomotion by decreasing the adhesion to water films in humid environments had been previously hypothesised to be the selective advantage of a haired shell [14,15]. However, the results of our experiments have shown that the opposite is true. The presence of hairs significantly increased the minimum force necessary to move shells over wet surfaces. Having thus shown that the initial hypothesis [14] is at least in this case not applicable, we propose an alternative: haired shells may confer an selective advantage by increasing the adhesion to the water film on the unstable, moving leaves of their feeding plants during foraging (Figure 5). Indeed, snails are mostly active during phases of high ambient humidity [27] when leaves are covered with a water film due to rain, fog or dew. This water film is usually in contact with the shell during locomotion (Figure 6). Observation shows that *Trochulus *species in moist habitats preferentially forage on large-leaved herbaceous plants like *Adenostyles*, *Urtica*, *Homogyne *or *Tussilago *[28]. Hence, falling off the leaf and needing to crawl up again to this feeding site (that can be one meter above ground) represents a considerable effort given the exceedingly costly and ineffective locomotion of land snails [29]. In dry habitats on the contrary, snail species avoid the hard plant matter typical for this habitat and preferentially feed on dead material lying on the ground [28,30], where a mechanism increasing shell adhesion offers no obvious advantage to its bearer. This interpretation is supported by the fact that phylogenetically distantly related haired species, such as *Helicodonta obvoluta *and *Isognomostoma isognomostoma*, are found in the same habitats and have in general similar life-styles [31]. However, as long as the positive effect of increased adherence to food plants on the individual fitness is not proven, this remains a hypothesis and does not preclude additional or even other adaptive functions of haired shells.

## Conclusion

The present comparative analysis suggested that hairs on the shell confer a selective advantage in humid habitats only and are thus lost in drier habitats. In other words, the variability of hairiness within the genus *Trochulus *could be explained in terms the loss of its adaptive function in a selectively different environment.

## Methods

### Taxon sampling

Analyses were undertaken on twelve of the about 15 currently recognised species presumed to belong to the genus *Trochulus s. str*. Chemnitz, 1786 (Hygromiidae, Stylommatophora). However, the exact number of existing species is not known, because the species limits of the widely distributed *T. hispidus *and *T. sericeus *on the one hand and *T. plebeius *and *T. striolatus *on the other are equivocal [19,20], the validity of several described taxa is disputed [18,32] and newly discovered species are not yet formally described (Pfenninger, unpublished data). Since initial analyses showed the existence of cryptic lineages, several populations for each of the putative species were sampled (Table 1). Four species from other genera of the subfamily Hygromiinae and two species of the family Helicidae were used as potential outgroups [33] (GenBank accession numbers AY546263, AY546343, AY546303, AY546284, AY546364, AY546324, AY546283, AY546363, AY546323, AY546291, AY546371, AY546331).

### DNA sequencing, lineage identification and phylogenetic analysis

Entire snails were crushed and vortexed in 10% w/v laundry detergent solution for storage at room temperature and tissue digestion [34]. For 78 individuals, a 512 bp segment of the cytochrome oxidase subunit I gene (COI) was amplified with PCR and sequenced. For selected individuals representing the major evolutionary lineages inferred in the previous analysis, a 362 bp fragment of the large subunit mitochondrial ribosomal gene (16S) and 509 bp of the internal transcribed spacer 1 (ITS-1) from the nuclear ribosomal cluster were additionally amplified and sequenced. An amount of 0.2 to 1 ng total DNA (quantified on a 1% agarose gel using a λ *Hind *III marker) were used as template in polymerase chain reaction (PCR). Specific PCRs were performed with the primers, amplification conditions and temperature profiles shown in Table 2. Primers were used for both specific PCR and subsequential automated direct sequencing. PCR products were purified using E.N.Z.A. Cycle Pure Kit (peqlab, Erlangen, Germany). Ten ng per sample were subjected to cycle sequencing using the ABI Prism Big Dye terminator kit (Perkin-Elmer, Norwalk, CT, USA). Sequencing reactions were electrophoresed on an ABI 377 automated DNA sequencer. In order to verify the results, gene products were sequenced in both directions and the two strands were aligned with SEQUENCE NAVIGATOR 1.0.1 (Perkin-Elmer, Norwalk, CT, USA). Sequences were deposited in GenBank under accession numbers DQ217794-DQ217831. The orthologous DNA sequences were initially aligned using the default settings of CLUSTAL X [35] and optimised by eye. The most likely models of sequence evolution and their parameters according to the Akaike information criterion were inferred for each DNA data partition using MODELTEST v. 3.4 [36]. In an initial analysis, we used the COI data set to identify evolutionary lineages. A 99.9% credible set of phylogenetic trees was estimated with the program MRBAYES [37] by sampling the tree space using a Metropolis coupled Monte Carlo Markov chain, implementing a TN+I+Γ model of COI sequence evolution (where TN denotes Tamura-Nei, Γ is the shape parameter of the gamma distribution and I the proportion of invariant sites). Initial runs as well as a posterior inspection of the likelihoods in the final run showed that a burn-in phase of 10,000 generations was largely sufficient for both analyses to allow the likelihood values to reach convergence. The chain was run for 10,000,000 generations and sampled every 100^th ^generation. An unrooted majority consensus tree was computed from the sampled trees, excluding the trees sampled in the burn-in phase. The procedure was repeated for the phylogenetic data set where the Markov chain was run with separate models of sequence evolution for each data partition (GTR (general time reversible)+I+G for 16S and TVM (transversional model)+ Γ for ITS-1). Outgroup status was assigned to *Helixaspersa *[33].

### Correlation of habitat humidity with shell hairiness

The direct estimation of humidity levels for sampling sites is difficult without long-term observation. However, the precipitation regime, habitat structure and vegetation at a sampling site can give clues on the degree of humidity experienced by the snails. For this behalf, five variables were recorded for all but one population belonging to *Trochulus s.str*. species. To characterise the microhabitat conditions, the mean light- and humidity indicator values [38] of the three most abundant herbaceous plant species at each sampling site were recorded (variables LIGHTIND and HUMIND). The evaporation regime is strongly influenced locally by the exposure to sun and wind, which was accounted for by characterising each sampling site as either i) entirely shadowed (2), partially or sometimes shadowed (1) and never shadowed (0) (variable SHADOW) and either ii) situated in a closed wood (2), open wood or forest edge (1) or not in a wood (0) (variable WOOD). Ultimately, the humidity conditions of a site depend on the precipitation in the area. As *Trochulus *species are active mainly during summer, we have recorded the average long-term precipitation from April to September (variable SUMMERPREC). This information was extracted from the climate layers with a spatial resolution of 0.5 min implemented in the computer program DIVA-GIS version 4.2 for the spatial analysis of biodiversity [39]. The variables were summarised in a principal component analysis (PCA).

For all *Trochulus s.str*. populations investigated, the presence or absence of hairs on the shell of at least 10 adult individuals was recorded. As the hairs may wear off during adulthood (although rarely completely), the lack of the typical hair pits in the fine sculpture of the shell was taken as evidence for their principal absence. The presence or absence of hairs of the respective populations was then plotted on the PCA ordination.

### Bayesian estimation of ancestral character states

In a first approach, we derived the posterior probability distribution of ancestral character states and their rate of change from 3000 trees sampled at random from the 99.9% credibility set of phylogenetic trees, using the Bayesian approach as implemented in the program MULTISTATEBAYES [40]. Applying an uninformative (uniform) prior on the rate parameter distribution, a Markov chain was run for 1,000,000 generations after it reached convergence. The estimated rate parameter ratio for both directions of character change as well as the reconstructed ancestral states for each internal node of the tree investigated was sampled every 200^th ^generation. This procedure estimates i) the probability that the ancestral node existed in the first place and ii) the probabilities of both character states at the respective node. These three probabilities sum up to 1, thus simultaneously taking phylogenetic and character mapping uncertainty into account. In a second approach, the most parsimonious number of character state changes was reconstructed for each of the 99.9% credibility set of phylogenetic trees using the ANCESTRAL STATE RECONSTRUCTION module in MESQUITE [41]. The different reconstructions were then weighted according to the posterior probability of the corresponding tree [42].

### Adhesion experiments

The minimum force necessary to move *Trochulus *shells (upwards oriented apex) with or without hairs over dry and wet, horizontal leaf surfaces was measured. For this behalf, we have chosen the largest species, *T. villosus*. It would have been desirable to use shells of other lineages as well, however, it was not possible to measure the force necessary to move smaller shells with the necessary accuracy. Twelve *T. villosus *shells were glued to thin nylon strings. The strings were led over a roll with a small aluminium basket fastened on the other end. Small weights were incrementally added to the basket until the shell began to slide. This was replicated five times for each shell on both water film covered and on dry surfaces. Then, the hairs were mechanically removed to obtain smooth shells and the procedure was repeated. For each condition, differences in minimum force needed to move the shells with or without hairs were tested for significance with an ANOVA design.

## Authors' contributions

MP designed the study, collected parts of the material, performed the analyses and drafted the manuscript. MH contributed to the samples and carried out part of the molecular work. DS also contributed to the samples and participated in the statistical analyses. AD contributed samples, participated in the design of the study and helped to draft the manuscript. All authors were involved in preparation of the manuscript and approved the final version.

**Table 3:** Primers used for specific PCR and direct sequencing, amplification conditions and temperature profiles.

Primer	Sequence	amplification conditions	temperature profile
COI universal [43]	5'-GGTCAACAATCATAAAGATATTGG-3' 5'-TAAACTTCAGGGTGACCAAAAAATCA-3'	total volume 25 μl with: 0.17 mM dNTPs 3 mM MgCl_2 _in 1 × PCR buffer 0.13 μM of each primer 1 unit Taq polymerase (Invitrogen)	1 cycle of 2.5 min at 94°C 40 cycle 30s at 90°C 1 min at 48°C 1 min at 72°C 1 cycle of 10 min at 72°C
16S universal [44]	5'-CGGCCGCCTGTTT ATCAAAAACAT-3' 5'-GGAGCTCCGGTTTGAACTCAGATC-3'	total volume 15 μl with: 0.1 mM dNTPs 2.5 mM MgCl_2 _in 1 × PCR buffer 0.2 μM of each primer 0.5 unit Taq polymerase (Invitrogen)	1 cycle of 2.5 min at 90°C 10 cycles of 50s at 92°C 30s at 44°C 40s at 72°C 36 cycles of 30s at 92°C 40s at 48°C 40s at 72°C 1 cycle of 3 min at 72°C
ITS-1 mollusc specific [45]	5'-TAACAAGGTTTCCGTAGGTGAA-3' 5'GCTGCGTTCTTCATCGATGC-3'	total volume 15 μl with: 0.3 mM dNTPs 2.5 mM MgCl_2 _in 1 × PCR buffer 0.18 μM of each primer 0.5 unit Taq polymerase (Invitrogen)	1 cycle of 3 min at 94°C 40 cycles of 30s at 92°C 30s at 52°C 1 min at 72°C 1 cycle of 5 min at 72°C
